# Endogenous dipeptidyl peptidase IV modulates skeletal muscle arteriolar diameter in rats

**DOI:** 10.14814/phy2.13564

**Published:** 2018-01-22

**Authors:** Leslie E. Neidert, Mohammed Al‐Tarhuni, Daniel Goldman, Heidi A. Kluess, Dwayne N. Jackson

**Affiliations:** ^1^ School of Kinesiology Auburn University Auburn Alabama; ^2^ Department of Medical Biophysics Schulich School of Medicine & Dentistry The University of Western Ontario London Ontario Canada

**Keywords:** Functional sympatholysis, gluteus maximus, neuropeptide Y, whey protein

## Abstract

The purpose of this study is to investigate that dipeptidyl peptidase IV (DPP‐IV) released from skeletal and vascular smooth muscle can increase arteriolar diameter in a skeletal muscle vascular bed by reducing neuropeptide Y (NPY)‐mediated vasoconstriction. We hypothesized that the effect of myokine DPP‐IV would be greatest in the smallest and least in the largest arterioles. Eight male Sprague Dawley rats (age 7–9 weeks; mass, mean ± SD: 258 ± 41 g) were anesthetized and the gluteus maximus dissected in situ for intravital microscopy analysis of arteriolar diameter of the vascular network. Computational modeling was performed on the diameter measurements to evaluate the overall impact of diameter changes on network resistance and flow distribution. In the first set of experiments, whey protein isolate powder was added to physiological saline solution, put in a heated reservoir, and applied to the preparation to induce release of DPP‐IV from the muscle. This resulted in an order‐dependent increase in arteriolar diameter, with the largest change in the 6A arterioles (63% more reactive than 1A arterioles; *P* < 0.05). This effect was abolished by adding the DPP‐IV inhibitor, Diprotin A. To test if the DPP‐IV released was affecting NPY‐mediated vasoconstriction, we applied NPY and whey protein, which resulted in attenuated vasoconstriction. These findings suggest that DPP‐IV is released from muscle and has a unique effect on blood flow, which appears to act on NPY to attenuate vasoconstriction. The findings suggest that DPP‐IV released from the skeletal or smooth muscle can alter muscle blood flow.

## Introduction

Neuropeptide Y_1‐36_, released by the sympathetic nerves, activates Y1 receptors on vascular smooth muscle and acts as a potent vasoconstrictor (Lundberg et al. 1985; Pernow et al. 1986; Zukowska‐Grojec 1995; Zukowska et al. 2003). However, NPY is enzymatically cleaved to become nonvasoconstricting neuropeptide Y_3‐36_ by the enzyme dipeptidyl peptidase IV (DPP‐IV or CD26) (Mentlein et al. 1993; Zukowska‐Grojec 1997; Mentlein 1999). DPP‐IV is present within the vasculature on the endothelium (Zukowska‐Grojec et al. 1998; Kitlinska et al. 2002; Zukowska et al. 2003; Pala et al. 2012) and the smooth muscle of the arterioles (Chung et al. 2010; Evanson et al. 2011, 2012). It is also expressed on the abluminal surface of blood vessels, where it truncates approximately 40% of the neuropeptide Y (NPY_1‐36_) released from the sympathetic neurons to become NPY_3‐36_ (Evanson et al. 2011, 2012). The effect of this enzyme on NPY bioavailability has a profound impact on the relative contribution of adrenergic versus nonadrenergic vasoconstriction in skeletal muscle microcirculation (Jackson et al., 2005a,b).

DPP‐IV is a ubiquitous, Type II cell surface glycoprotein that exists in two basic forms, membrane bound and soluble, and exerts its actions primarily through its peptidase activity and interaction with extracellular matrix components. Membrane‐bound DPP‐IV is present in high levels on the luminal and abluminal side of the arterioles and venules, in the proximal renal tubular cells of the kidneys, and in T cells, whereas soluble DPP‐IV is found dissolved in the blood and interstitial fluid (Mentlein 1999). In earlier work, it was thought that the pool of DPP‐IV that modifies the sympathetic neurotransmitter, NPY, was exclusively membrane bound and located on the adventitial side of the vasculature (Chung et al. 2010; Evanson et al. 2011, 2012). As well, past research illustrates that DPP‐IV activity can be altered chronically by estrogen (Jackson et al., 2005a,b). However, more current evidence suggests that skeletal muscle (Raschke et al. 2013; Neidert et al. 2016) and smooth muscle (Rohrborn et al. 2014) may release soluble DPP‐IV, as a myokine, into the interstitial space. These findings opened the possibility that NPY could be modified by soluble as well as membrane‐bound DPP‐IV.

Previous in vitro work by Neidert et al. (2016) revealed that DPP‐IV release from skeletal muscle cells can be stimulated by adding whey protein to cell culture media at a concentration that mimics the endogenous protein concentration measured in skeletal muscle interstitial fluid. This effect of whey protein is caused by matrix metalloproteinases (MMPs) in the whey protein mixture (Raulo et al. 2002; Lubetzky et al. 2010). The MMPs cleave membrane‐bound DPP‐IV out of the muscle membrane to create soluble DPP‐IV (Hooper et al. 1997; Rohrborn et al. 2014). While the application of whey protein by this method is not physiologically relevant, it is an experimental model that produces large changes in DPP‐IV activity that are not complicated by the effects of muscle contraction. This allows direct exploration of the functional significance of the DPP‐IV release on NPY‐mediated vasoconstriction. Therefore, the purpose of the current project was to investigate the possibility that DPP‐IV released from skeletal muscle can alter skeletal muscle microvascular resistance by reducing NPY‐mediated vasoconstriction. We hypothesized that the effect of myokine DPP‐IV would be greatest in the smallest arterioles and least in the largest arterioles. This hypothesis is based on work by Al‐Khazraji et al. (2015) demonstrating that NPY‐mediated vasoconstriction is greatest in the distal arterioles and least in the proximal arterioles.

## Methods

### Ethical approval

All experimental protocols were approved by the University of Western Ontario Council on Animal Care (AUP#2012‐018). During the experiment, the rats were anesthetized via i.p. injection with *α*‐chloralose (80 mg·kg^−1^) and urethane (500 mg·kg^−1^) dissolved in sterile ddH_2_O. All efforts were made to minimize the number of animals used and their suffering. At the end of the experiment, the anesthetized rat was euthanized via an overdose (i.p.) of *α*‐chloralose and urethane cocktail mix and cervical dislocation.

### Animal care and use

Eight male Sprague Dawley rats (age: 7–9 weeks; mass: 258 ± 41 g) were purchased from Charles River Laboratories (Saint‐Constant, Quebec, Canada) and housed in the University of Western Ontario animal care facilities for at least a week prior to the study. Rats were kept on a 12:12‐h light/dark cycle at standard room temperature and were provided food and water ad libitum.

### Anesthesia and skeletal muscle preparation

Methods are described in more detail in a previously published manuscript (Al‐Khazraji et al. 2012). Briefly, the rat was anesthetized via i.p. injection, with *α*‐chloralose (80 mg·kg^−1^) and urethane (500 mg·kg^−1^) dissolved in sterile ddH_2_O. The fur of the neck and lower back region was shaved, the animal was tracheotomized (to facilitate spontaneous breathing), and the right jugular vein was cannulated to maintain a constant infusion of anesthetic to the animal (*α*‐chloralose: 8–16 mg/kg/h, urethane: 50–100 mg/kg/h). Finally, the left common carotid artery was cannulated (PE‐50 tubing) to allow for recording of arterial blood pressure via the amplified signal of a pressure transducer using a PowerLab system (model ML118 PowerLab Quad Bridge Amplifier; model MLT0699 BP Transducer, ADInstruments, Colorado Springs, CO, USA).

A customized temperature‐controlled platform was utilized to maintain body temperature at 37°C, monitored by rectal probe. Under microscopic guidance, the gluteus maximus (GM) muscle was dissected along its rostral and caudal borders and along the spine and carefully reflected away from the rat. The muscle flap was spread evenly on a transparent pedestal (Sylgard 184; Dow Corning, Midland, MI, USA) to approximate in situ dimensions and pinned to secure edges. Exposed tissue was continuously superfused with bicarbonate‐buffered PSS (flow rate: 4–5 mL·min^−1^; 35°C at pH of 7.4; 137 mmol/L NaCl, 4.7 mmol/L KCl, 1.2 mmol/L MgSO_4_, 2 mmol/L CaCl_2_, and 18 mmol/L NaHCO_3_), equilibrated with 5% CO_2_/95% N_2_ gas.

### Intravital video microscopy

Once microsurgical preparation was complete, the preparation was moved to the stage of an intravital microscope (Olympus BX51; Olympus, Tokyo, Japan), where it equilibrated for 30 min. During equilibration, the arterial network was scanned for experimental sites for observation. Microvessels were imaged under bright‐field transillumination using a long working condenser (NA = 0.80) and a long working distance water immersion objective (Olympus LUMPLFL: 10× NA = 0.30; depth of field ~9 *μ*m) with illumination from a 100 W halogen light source. The optical image was coupled to an EMCCD camera (Rolera EMC2; Qimaging, Surrey, BC, Canada), viewed using specialized imaging software (Metamorph, Version 7.6; Molecular Devices, Inc., Sunnyvale, CA, USA), and stored on a hard drive for offline analysis. The baseline interval vessel lumen diameter was recorded after equilibration, and arterioles were tested for oxygen sensitivity by increasing the superfusate O_2_ from 0% to 21% (5% CO_2_, balance N_2_) for 5 min and recording the arteriolar diameters (Table 1). After O_2_ sensitivity was established, noted by a stable reduction in arteriolar diameter, the 5% CO_2_/95% N_2_ was restored for the duration of the experiment. At the end of each experiment, maximal arteriolar dilation to a topical application of sodium nitroprusside (SNP; 10^−5^ mol/L) was carried out. Bright‐field video (.tiff) images were collected (17fps) under bright‐field illumination for offline analysis of internal vessel lumen diameters using ImageJ (NIH, Bethesda, MD, USA).

**Table 1:** Arteriolar network branches for each animal

Animal	1A	2A	3A	4A	5A	6A
1	1	2	4	8	10	2
2	1	2	5	6	6	8
3	1	2	4	4	4	2
4	1	2	4	4	4	2
5	1	2	4	6	6	6
6	1	2	4	4	6	4
7	1	2	4	6	6	8
8	1	2	4	6	8	4
Total	8	16	33	44	50	36

Each animal is treated as *n* = 1, and the measurements from each branch is averaged to be the value for that individual animal.

### Preparation and delivery of experimental solutions

All working solutions of drugs were prepared the morning of experimentation and were dissolved in gassed and warmed PSS solution All solutions were pregassed prior to preparation and were gassed continuously in the reservoirs to ensure continuous and homogenous gas dispersion. Two water‐jacketed reservoirs (Radnoti LLC, CA, USA) were used. One provided warmed and gassed PSS via gravity feed from a larger reservoir, and the second provided the warmed and gassed experimental solutions, which were added to the reservoir. All solutions were applied topically to the muscle preparation via gravity feed at a rate of 5 mL/min and a temperature of 35°C.

### Experimental protocols

Using a repeated measures design, protocols 1 and 2 were randomized and counterbalanced (*n* = 8 rats).

#### Protocol 1: In situ endogenous DPP‐IV release from skeletal muscle and its impact on baseline arteriolar resistance

We have previously shown that whey protein promotes the release of endogenous DPP‐IV from skeletal muscle cells in vitro (Neidert et al. 2016). Thus, using the in situ GM experimental preparation, the objectives for this set of experiments were to (1) test if topical application of whey protein isolate augments endogenous DPP‐IV release from skeletal muscle in situ and (2) test the impact of augmented endogenous DPP‐IV release on arteriolar resistance.

Pure whey protein isolate powder (WPI; 100 mmol/L; Glanbia Nutritionals Inc., Fitchburg, WI, USA), mixed into solution with PSS, was added to the heated reservoir and applied to the preparation. Arteriolar responses were recorded when the diameters stabilized (within 3 min) and remained static for a minimum of 5 min. Following a baseline scan of the microvascular tree and at the end of each intervention, 1 mL of effluent PSS was collected from the preparation by briefly stopping the vacuum to allow the solution to collect on the preparation. This sample of PSS was stored (−80°C) for future analysis of DPP‐IV activity.

To test if arteriolar responses to the topical application of WPI were due to augmented DPP‐IV release a DPP‐IV inhibitor, Diprotin A (4 *μ*mol/L; Sigma Aldrich, St Louis, MO), was added to the PSS/WPI solution. Arteriolar responses were imaged, recorded, and stored for offline analysis (see Experimental data collection below). Upon completion of microvascular imaging, a 1‐mL sample of effluent PSS was collected from the preparation for the analysis of DPP‐IV activity and, immediately after, the control PSS drip was restarted. Once microvascular responses returned to baseline and were stable (~10 min) the next protocol was started. In three experiments, an additional control experiment was performed. As a negative (protein) control, in a subgroup of rats (*n* = 3), bovine serum albumin (BSA; Sigma Aldrich, USA) was substituted for whey protein in the superfusate at the same concentration (100 mmol/L).

#### Protocol 2: In situ endogenous DPP‐IV release and its impact on NPY‐mediated arteriolar constriction

NPY (10^−10^mol/L; Tocris Bioscience, USA) was added to PSS and applied to the preparation. Arteriolar responses were recorded, solution was collected, and the preparation was returned to baseline with control PSS before the WPI + NPY solution was added. NPY was added to the WPI solution at the same concentration as above. This solution was then added to the reservoir suspended over the muscle preparation. Responses and solution were collected, followed by the addition of DPP‐IV inhibitor in the same manner as Protocol 1.

### Experimental data collection

Arteriolar responses at different branch orders (from 1A to 5A, and 6A when available) were recorded for each experimental solution. Once all experimental protocols were complete, the tissue was superfused with PSS, and arteriolar diameters were restored to baseline measures. Finally, maximum diameter responses to SNP were recorded. During each scan, continuously branching sets of arteriolar trees were scanned and imaged in descending order, followed by a scan in ascending order to confirm that the network was stable. The total imaging/scan time was under 30 sec. For all scans, the arteriolar responses at the beginning and end of imaging did not differ, indicating no changes occurred in the responses during the scanning period.

### DPP‐IV activity assay

The DPP‐IV activity of the solution from the preparation was determined in order to evaluate the amount of DPP‐IV released from the muscle. DPP‐IV Glo protease assay kit (Promega, Madison, WI) was used following the manufacturer's instructions. DPP‐IV activity was expressed as relative light units (RLU).

### Computational analysis

To estimate the overall network hemodynamics consequences resulting from diameter changes, experimental findings from a single, complete arteriolar network were incorporated into a previously described and validated two‐phase (RBCs and plasma) steady‐state continuum blood flow model (Pries et al. 1990, 1994; Goldman and Popel 2000, 2001; Al‐Khazraji et al. 2015) (Fig. 3). The arteriolar network geometry and topology were determined from an intravital video microscopy photomontage, and consisted of 145 unbranched vessels with a single inlet vessel (1A arteriole) and 73 outlet vessels (3A–8A arterioles). Baseline diameters ranged from 128 *μ*m for the inlet vessel to an average of 16.1 ± 9.8 *μ*m (mean ± SD) for the outlet vessels. For each experimental condition, arteriolar diameter measurements were made approximately every 150 *μ*m throughout the network considered, and these diameter values were used as inputs for each simulation. Blood flow was simulated using a fixed pressure drop of 9 mmHg from inlet to all outlet vessels, and the inlet discharge hematocrit was set to 0.42. The findings from the model were used to estimate the changes in total blood flow of the network (*Q*_total_), network resistance (*R*_tot_), and RBC flow heterogeneity (coefficient of variation of RBC volume flow) arising from the role of DPP‐IV in the branching arteriolar network.

### Statistical analysis

Experimental data are presented as means ± standard deviations, unless otherwise stated. Arteriolar network information is presented in Table 1. Each animal represents *n* = 1, and each set of branches for the individual animal are averaged together as the value for that animal. Constriction responses were calculated as changes from the baseline diameter (% constriction = [D_Baseline_ − D_Response_]/D_Baseline_ × 100%). Between‐order reactivity was presented as the percent change difference ([%Constriction_A_ − %Constriction_B_]/%Constriction_A_ × 100%). For each experimental condition, a one‐way ANOVA was used to determine the statistically significant effect of arteriolar order on the amount of constriction. Tukey's post hoc analysis was performed in the case of statistical significance. In order to determine the differences between the experimental conditions, a two‐way repeated measures ANOVA was performed for Protocols 1 and 2, with *arteriolar order* and *treatme*nt as the two variables. Bonferroni post hoc test was used if the results of the analysis were statistically significant. The *α*‐level for all analyses was set at 0.05 for statistical significance.

## Results

Animals exhibited stable and normal mean arterial pressure (98 ± 3 mmHg) and heart rate (379 ± 21 bpm) throughout the experimental period.

### Baseline testing

Bubbling the preparation with oxygen resulted in a −15.8 ± 6.2% change in diameter from baseline (*P* < 0.0001) in all animals. At the end of the experiments, sodium nitroprusside (SNP) was added and resulted in an 18.9 ± 18.2% increase in diameter from baseline (*P* < 0.0001) in all animals. Summary data for the baseline arteriolar diameters and the responses to oxygen and SNP are presented in Table 2.

**Table 2:** Summary values for luminal diameters for five arteriolar orders

Arteriolar order (*n* = 8)	Baseline diameter (*μ*m)	O_2_ response (*μ*m)	SNP response (*μ*m)
1A	111.1 ± 11.4	−16.4 ± 8.4	22.4 ± 20.7
2A	85.5 ± 6.0	−13.4 ± 4.9	22.0 ± 10.3
3A	61.1 ± 4.8	−7.0 ± 8.4	16.5 ± 7.1
4A	45.7 ± 3.5	−6.4 ± 5.7	13.8 ± 5.9
5A	35.1 ± 6.2	−2.4 ± 8.5	13.8 ± 5.6

Responses were calculated as changes from the baseline diameter (D_Response_ − D_Baseline_) and reported as mean ± SD.

### Protocol 1: Whey protein data

**Figure 1:**
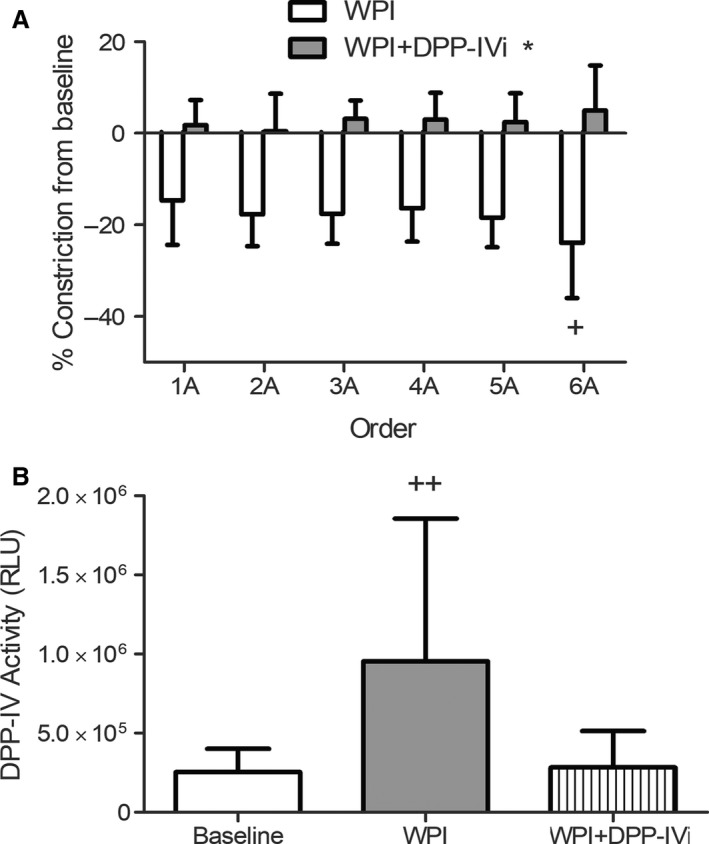
Whey protein attenuates basal vasoconstriction via release of DPP‐IV. (A) The addition of whey protein to the topical solution resulted in a decrease in vasoconstriction. The largest change was in the 6A arterioles (^+^ *P* < 0.05 different from 1A and 4A). When the DPP‐IV inhibitor, Diprotin A (DPP‐IVi), was added, the effect was abolished (*P < 0.05; all orders compared to WPI condition). (B) The topical solution bathing the muscle and arteriolar network was collected and analyzed for DPP‐IV activity. DPP‐IV activity was significantly increased by whey protein compared to baseline (*P* < 0.05).

There was an arteriole order‐dependent effect of whey protein isolate (WPI) on arterial constriction (*n* = 8; Fig. 1A). All orders of arterioles dilated, with the maximum response at 6A arterial order. 6A arterioles were 63% more reactive than 1A and 46% more reactive than 4A arterioles (*P* < 0.05). Dilation was prevented by the addition of DPP‐IV inhibitor (*P* < 0.05) across all orders. As a control, we used BSA in the same concentration as whey protein to assess the likelihood that it was the protein concentration alone that caused the effect seen with whey protein. BSA resulted in no change in vessel diameter from baseline (0.2 ± 1% change in diameter from baseline; *n* = 3). We measured the DPP‐IV activity in the effluent from the preparation and found a large increase in DPP‐IV activity with whey protein (baseline: 251,868 ± 148,812 RLU vs. WPI: 953,886 ± 902,469 RLU; *P* < 0.05; *n* = 8). This increase was abolished with the DPP‐IV inhibitor, Diprotin A (Fig. 1B).

### Protocol 2: Whey protein and NPY data

**Figure 2:**
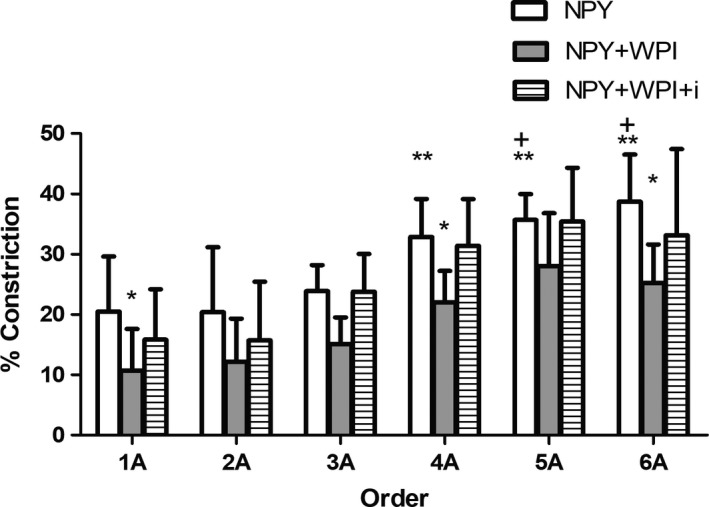
Neuropeptide Y‐mediated vasoconstriction with whey protein. Neuropeptide Y resulted in vasoconstriction in all orders, but the greatest constriction was in the 4A, 5A, and 6A arterioles compared to the 1A and 2A arterioles (**P < 0.05). The constriction via 3A arterioles was less than that which occurred in 5A and 6A arterioles (^+^ *P* < 0.05). The addition of whey protein attenuated neuropeptide Y‐mediated vasoconstriction in 1A, 4A, and 6A arterioles (*P < 0.05). This effect was abolished by adding the DPP‐IV inhibitor, Diprotin A (DPP‐IVi).

There was an arteriole order‐dependent effect of NPY on arterial constriction (*n* = 8; Fig. 2). Average arterial constriction was greatest at 6A, which decreased with ascending arteriole order (6A, 5A, 4A>2A, 1A; 6A, 5A>3A; *P* < 0.05). When whey protein and NPY were present, constriction was attenuated across all orders (only 1A, 4A, and 6A were significantly decreased; *P* < 0.05). There was an order‐dependent effect, with 4A, 5A, and 6A having a greater decrease than 1A and 2A arterioles (*P* < 0.05). This attenuation was rescued with the addition of DPP‐IV inhibitor (DPP‐IVi), for which the NPY + WPI + DPP‐IVi condition was not significantly different from NPY alone.

### Computational analysis

**Figure 3:**
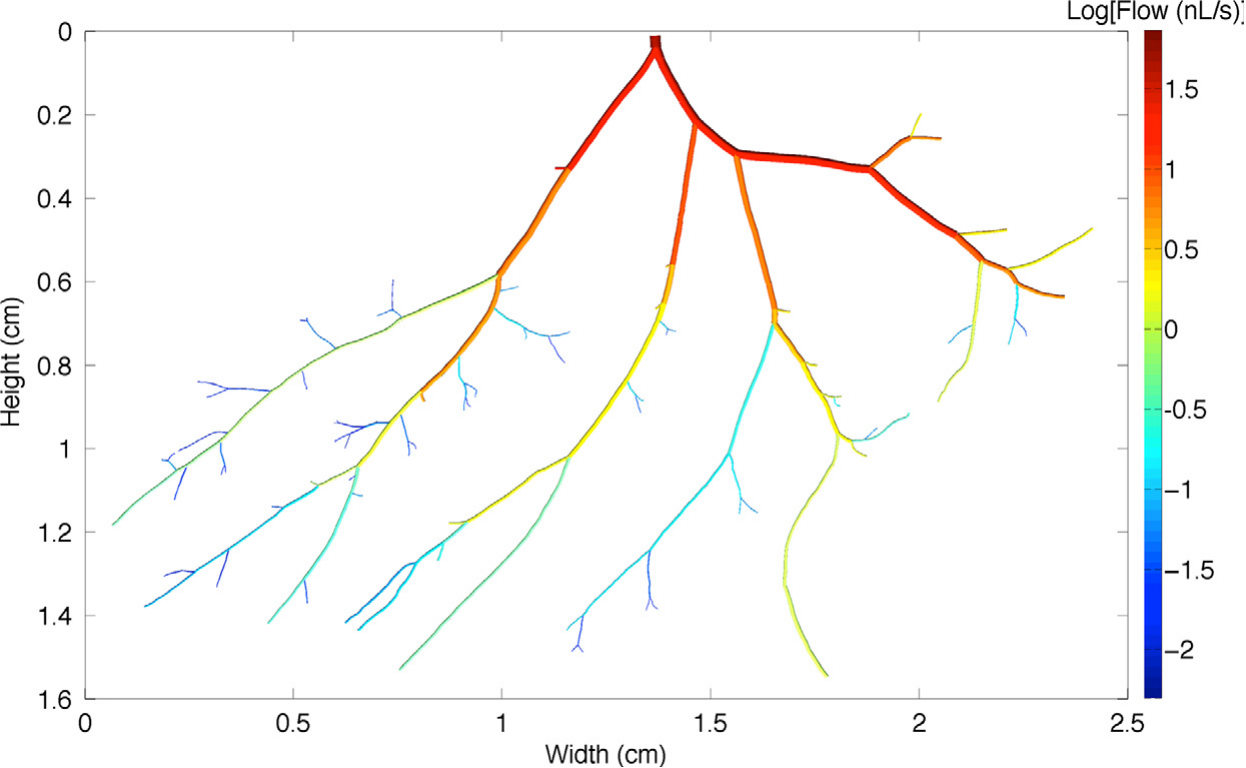
Reconstructed arteriolar network used in computational modeling. This figure shows the structure of the network used including baseline (control) diameters. The network is color coded based on calculated blood flow rate through vessel segments under control conditions, with red indicating high flow and dark blue indicating low flow.

The network used for blood flow calculations had diameter changes from baseline similar to the mean results in Figure 1. In particular, 1A–6A vessels in this network had a mean constriction to WPI of −22.4% (1A: −28.3%, 2A: −32.3%, 3A: −24.2%, 4A: −26.5%, 5A: −14.7%, 6A: −8.5%), and a mean constriction to WPI + DPP‐IVi of −6.5% (1A: −13.1%, 2A: −18.2%, 3A: −4.5%, 4A: −3.4%, 5A: −1.0%, 6A: +1.2) (Fig. 3).

**Figure 4:**
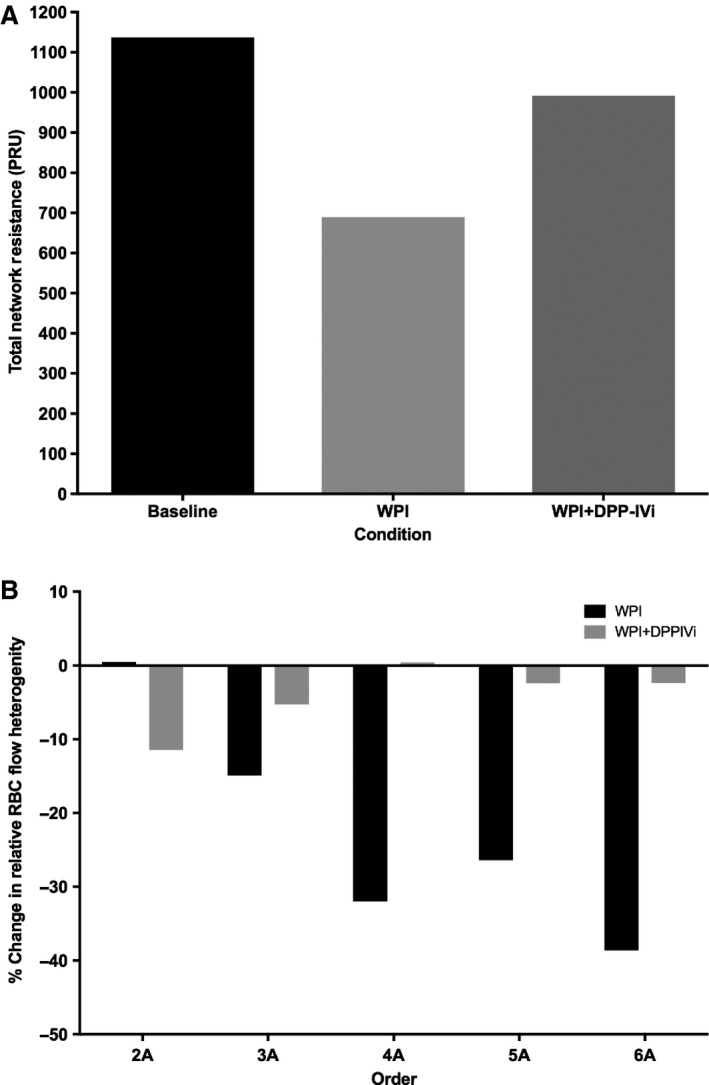
Changes in network blood flow properties with whey protein (WPI) and the DPP‐IV inhibitor, Diprotin A (WPI + DPP‐IVi). (A) Network resistance was reduced by whey protein and recovered by Diprotin A. (B) Whey protein (WPI) resulted in a decrease in relative red blood cell heterogeneity in the 3A–6A arterioles. This effect was abolished by Diprotin A (WPI + DPP‐IVi).

Calculated total blood flow (*Q*_tot_) in the network used for modeling increased by approximately 64% from baseline (131.9 nL/sec) to WPI (217.6 nL/sec). *Q*_*tot*_ was restored to within 15% of baseline values (151.3 nL/sec) under WPI + DPP‐IVi. Similarly, total network resistance was estimated to be 1136.9 PRU at baseline. With the addition of WPI, total network resistance (*R*_tot_) decreased by approximately 61% from baseline values (689.3 PRU). (Fig. 4A). With the addition of DPPI‐IVi, *R*_tot_ increased back to within 13% of baseline values (991.6 PRU).

RBC volume flow heterogeneity was estimated using the coefficient of variation (standard deviation divided by mean) of RBC volume flow within vessels of each topological order in the network. For WPI and WPI + DPP‐IVi, the percent change in RBC flow heterogeneity for each order was calculated with respect to the corresponding baseline levels of heterogeneity. With the addition of WPI, RBC flow heterogeneity decreased from baseline values, particularly at topological orders 3 and higher (2A: −1.3%, 3A: −14.9%, 4A: −32.0%, 5A: −28.4%, 6A: −38.6%) (Fig. 4B). Following the addition of DPP‐IVi, RBC flow heterogeneity in 3A and higher order vessels was restored to values similar to baseline levels (2A: −11%, 3A: −5.3%, 4A: 0.4%, 5A: −2.4%, 6A: −2.4%).

## Discussion

In the current study, the vascular responses of the rat gluteus maximus arteriolar network to the myokine, DPP‐IV, were investigated. Using whey protein as a stimulus for release of DPP‐IV from skeletal muscle, we found that DPP‐IV released from the local muscle elicited a robust increase in arteriolar diameter, with responses being greatest in small bore distal arterioles. This effect was abolished by a DPP‐IV inhibitor and supports the postulate that DPP‐IV is released, in a myokine fashion, from skeletal muscle, resulting in profound effects on baseline microvascular network resistance, blood flow, and RBC distribution (as noted by changes in network RBC flow heterogeneity). When NPY was added to the preparation, it caused an arteriole order‐dependent decrease in diameter, which was attenuated by the addition of whey protein. The attenuation was largely eliminated by the addition of a DPP‐IV inhibitor. These findings suggest that the myokine, DPP‐IV, acts on NPY to attenuate vasoconstriction. Although the method of inducing release of DPP‐IV in this study was not physiological (topically applied whey protein), the findings are consistent with the notion that DPP‐IV released from the skeletal or smooth muscle can alter muscle blood flow and may be a previously unrecognized mechanism for functional sympatholysis.

### Whey protein stimulated DPP‐IV release

Previous research showed that DPP‐IV could be released from myocyte cell culture (Raschke et al. 2013). Further work by the Kluess Lab (Neidert et al. 2016) demonstrated that when whey protein was added to myocyte cell culture, the myocytes release DPP‐IV into the interstitial space. DPP‐IV can also be released from smooth muscle cells in culture (Rohrborn et al. 2014), therefore, in the current study, we cannot distinguish between the type of muscle that released the DPP‐IV. Regardless of the location of release, the functional significance of muscle release of DPP‐IV was unknown. The current study is the first study to demonstrate that the myokine DPP‐IV is capable of altering blood flow, likely via attenuating NPY‐mediated vasoconstriction via cleavage of NPY_1‐36_ into its nonvasoconstricting form (Mentlein 1999; Zukowska et al. 2003). NPY is released at rest (Buckwalter et al. 2005; Jackson et al. 2005a) and acts as a potent vasoconstrictor.

In these data, when whey protein was topologically applied to the gluteus maximus preparation, an increase in arteriolar diameter was observed with the 6A arterioles exhibiting the largest effect and 1A arterioles the smallest effect. The arteriole order‐dependent manner of response is consistent with the findings of Evanson et al. (2011), who demonstrated greater DPP‐IV activity in the 1A arteriole of the gastrocnemius when compared to the larger femoral artery. This is also consistent with the evidence showing that NPY‐mediated vasoconstriction and presumably the greatest Y1 receptor density occurs in the smaller arterioles (Al‐Khazraji et al. 2015). Our findings were further strengthened when the inhibition of DPP‐IV via Diprotin A attenuated the increase seen in the arteriolar diameter in response to whey protein. Diprotin A acts by binding to the active site of DPP‐IV and preventing its action on other substrates (Hiramatsu et al. 2004). Therefore, DPP‐IV may still be released, but it is rendered inactive (and not measurable by the assay) by Diprotin A. Diprotin A does not exclusively target soluble DPP‐IV, but will also inhibit membrane‐bound DPP‐IV on the abluminal surface of the arterioles (Jackson et al. 2005b; Evanson et al. 2011, 2012). Therefore, part of the effect seen with Diprotin A may be from the membrane‐bound DPP‐IV.

We also ran an albumin control in three animals to test for a general protein concentration effect. Bovine serum albumin in the same concentration as the whey protein did not result in a change in arteriolar diameter. Whey protein contains MMPs, which were demonstrated to cause shedding of DPP‐IV in human smooth muscle cells and adipocytes (Rohrborn et al. 2014). This finding was extended to skeletal muscle myocytes by Neidert et al. (2016), who showed that blocking the MMPs present in whey protein resulted in reduced DPP‐IV shedding from the myocyte membrane. This is significant because MMPs can increase with hypoxia (Rohrborn et al. 2014) and with hyperglycemia (Death et al. 2003; Chang and Vivian Yang 2013) and exercise (Schild et al. 2016), and may decline as women age (Nascimento Dda et al. 2016).

To further support the DPP‐IV inhibitor data, we also measured DPP‐IV activity in the topical solution bathing the muscle, showing that DPP‐IV was released from the preparation. Previous work showed that DPP‐IV is a myokine (Raschke et al. 2013) and can be released from cultured skeletal muscle cells (Neidert et al. 2016). In the current study, we have expanded this by demonstrating that DPP‐IV can also be released from intact mature muscle and, possibly, vascular smooth muscle in vivo. The most likely route of release with whey protein is shedding of membrane‐bound DPP‐IV via MMP‐2 and MMP‐9 (Rohrborn et al. 2014; Neidert et al. 2016).

### The effect of the myokine DPP‐IV on NPY‐mediated vasoconstriction

In the second set of experiments, we added NPY to the topical solution to cause constriction of the arteriolar network. This concentration of NPY was previously used by Al‐Khazraji et al. (2015) and was estimated to be at approximately EC_50_ in the smallest arterioles. Our data support the findings of these earlier investigations that give evidence for the order dependence of NPY‐mediated vasoconstriction (Al‐Khazraji et al. 2015). This response is attributed to larger numbers of receptors in the smaller arterioles rather than increased concentrations of NPY at those locations.

When NPY was applied in combination with whey protein, the vasoconstriction response was attenuated by approximately 19% (all orders), supporting the idea that the release of DPP‐IV was acting to truncate NPY_1‐36_ to NPY_3‐36_, which alters the neuropeptides ability to bind to Y1 receptors and cause vasoconstriction (Mentlein 1999). Previous measurements of the kinetics of DPP‐IV found that the Kcat/Km for the reaction with NPY was 7.6 × 10^5^ M^−1^s^−1^ (Lambeir et al. 2001). This is in agreement with work by Evanson et al. (2011) showing that NPY elicits vasoconstriction in isolated arterioles and arteriolar networks. However, this is the first study to demonstrate that DPP‐IV released from muscle can cause this effect. If this effect also occurs during exercise, myokine DPP‐IV may play an important role in functional sympatholysis during exercise.

### Blood flow modeling

Al‐Khazraji et al. (2015) used a computational model of blood flow to determine the impact on arteriolar network resistance and RBC distribution of sympathetic receptor activation based on a reconstructed baseline network and mean changes in diameter approximated as functions of vessel order and baseline diameter. In the present work, our blood flow modeling used a single reconstructed arteriolar network with all vessel diameters measured in the same network under the three experimental conditions considered. Thus, although the experimental data used in the model might not exactly match the physiological effects of DPP‐IV, no assumptions were made about how diameter changes varied across the network, which allowed us to directly integrate measured diameter changes to estimate the functional consequences of DPP‐IV release and inhibition. This showed the degree to which DPP‐IV decreased overall flow resistance of the arteriolar tree in our experiments, and also showed that DPP‐IV substantially decreased the heterogeneity of RBC distribution to higher order (i.e., terminal) arterioles. Together, these two results indicate that DPP‐IV has a major positive effect on muscle perfusion and should enhance blood tissue mass transport including the delivery of oxygen.

### DPP‐IV inhibition

In the last 20 years, the research on DPP‐IV has increased, but most of this research looks at the benefits of DPP‐IV inhibition. The inhibition of DPP‐IV is a popular treatment for diabetic individuals because DPP‐IV cleaves glucagon‐like peptide 1 (GLP‐1) into its inactive parts and prevents GLP‐1‐mediated insulin release (Holst and Deacon 2005; Drucker and Nauck 2006). Many studies showed the effectiveness of DPP‐IV inhibiting drugs (Drucker and Nauck 2006; Green et al. 2006; Nauck et al. 2016), and it has now grown to include treatment for other conditions (Avogaro and Fadini 2014; Forst et al. 2016; Saboo et al. 2016). However, the current study demonstrates the potential physiological benefits of DPP‐IV to skeletal muscle blood flow. Therefore, it is possible that DPP‐IV inhibition could have negative effects in certain parts of the body. Ayaori et al. (2013) showed that patients with Type 2 diabetes on DPP‐IV inhibitors saw unfavorable reactions to flow‐mediated dilation even though there were positive results in their diabetic status. This is further supported by another study showing DPP‐IV‐deficient rats had inferior endothelial function, which suggests DPP‐IV is necessary for maintaining vascular function (Sun et al. 2013).

In one study by Shah et al. (2011), inhibition of DPP‐IV on preconstricted human umbilical vein endothelial cells (HUVECs) caused relaxation of the vasculature mediated by endothelium‐derived NO. A more recent study shows *db/db* mice given a DPP‐IV inhibitor increased NO availability, improving vascular function (Solini et al. 2016). In the present study, however, all treatments were applied via the abluminal side, causing the interaction between the endothelial cells and treatments to be limited, if not completely prevented. DPP‐IV is a large protein (~110 kDa), and likely does not pass through the vessel walls easily, suggesting that the vascular responses were likely due to abluminal signaling and not intraluminal pathways via the endothelium.

## Conclusion

This is the first study to demonstrate that DPP‐IV released by muscle causes an attenuation of NPY‐mediated vasoconstriction. This effect is largest in the smallest arterioles and has a large effect on modeled muscle blood flow as estimated by computational modeling. Therefore, it is possible that myokine DPP‐IV may play an important role in attenuating NPY‐mediated vasoconstriction during exercise.

## Conflict of Interest

There are no competing interests to report.
